# A multiple case history and systematic review of adoption, diffusion, implementation and impact of provincial daily physical activity policies in Canadian schools

**DOI:** 10.1186/s12889-015-1669-6

**Published:** 2015-04-15

**Authors:** Dana Lee Olstad, Elizabeth J Campbell, Kim D Raine, Candace IJ Nykiforuk

**Affiliations:** Centre for Physical Activity and Nutrition Research, School of Exercise and Nutrition Sciences, Deakin University, 221 Burwood Highway, Burwood, VIC 3125 Australia; School of Public Health, 3–300 Edmonton Clinic Health Academy, University of Alberta, 11405 87 Avenue, Edmonton, AB T6G 1C9 Canada

**Keywords:** Physical activity, Policy, Diffusion, Adoption, Implementation, Impact, Systematic review, Children, School

## Abstract

**Background:**

Few children meet physical activity (PA) recommendations, and are therefore at increased risk for overweight/obesity and adverse health outcomes. To increase children’s opportunities for PA, several Canadian provinces have adopted school-based daily PA (DPA) policies. It is not clear why some jurisdictions have adopted DPA policies, and others have not, nor whether these policies have been implemented and have achieved their intended outcomes. The purpose of this study was to understand the processes underlying adoption and diffusion of Canadian DPA policies, and to review evidence regarding their implementation and impact.

**Methods:**

We adopted a multiple case history methodology in which we traced the chronological trajectory of DPA policies among Canadian provinces by compiling timelines detailing key historical events that preceded policy adoption. Publicly available documents posted on the internet were reviewed to characterize adopter innovativeness, describe the content of their DPA policies, and explore the context surrounding policy adoption. Diffusion of Innovations theory provided a conceptual framework for the analyses. A systematic literature search identified studies that had investigated adoption, diffusion, implementation or impact of Canadian DPA policies.

**Results:**

Five of Canada’s 13 provinces and territories (38.5%) have DPA policies. Although the underlying objectives of the policies are similar, there are clear differences among them and in their various policy trajectories. Adoption and diffusion of DPA policies were structured by the characteristics and capacities of adopters, the nature of their policies, and contextual factors. Limited data suggests implementation of DPA policies was moderate but inconsistent and that Canadian DPA policies have had little to no impact on school-aged children’s PA levels or BMI.

**Conclusions:**

This study detailed the history and current status of Canadian DPA policies, highlighting the conditional nature of policy adoption and diffusion, and describing policy and adopter characteristics and political contexts that shaped policy trajectories. An understanding of the conditions associated with successful policy adoption and diffusion can help identify receptive contexts in which to pioneer novel legislative initiatives to increase PA among children. By reviewing evidence regarding policy implementation and impact, this study can also inform amendments to existing, and development of future PA policies.

**Electronic supplementary material:**

The online version of this article (doi:10.1186/s12889-015-1669-6) contains supplementary material, which is available to authorized users.

## Background

Children who engage in 60 minutes of moderate-to-vigorous physical activity (MVPA) daily are less likely to be overweight/obese and to exhibit risk factors for chronic disease [1]. For this reason, Health Canada [2,3] and other national governments [4] recommend that children accumulate at least 60 minutes of MVPA daily. Many Canadian children are not sufficiently active to achieve health benefits, however, as only 8% of boys and 4% of girls aged 6–17 meet Canadian physical activity (PA) guidelines [5]. Youth report that many of the barriers they face with respect to PA are environmental, in that they perceive having insufficient time, opportunity and resources to be more physically active [6,7].

School-based initiatives provide an opportunity to reach children at a critical time point, as they are developing attitudes and behaviors that may influence their future health [8]. Schools are particularly suited for PA interventions because children already spend a substantial amount of time there, they have existing PA-related infrastructure, and they require most students to engage in some amount of PA through Physical Education (PE) courses. However, although children who participate in PE are more physically active than their counterparts [9-12], Canadian children spend < 15% of their PE time in MVPA [13], and cost-cutting measures have reduced the overall quality and quantity of PE provided [14-18]. In high schools, enrollment in PE has declined over time [19] and declines at higher grades to levels as low as 29% in some cases [9,16,19,20]. Similar trends have been identified in the US, where children are spending more time in sedentary, academic pursuits and less time in PE and recess as a result of legislation associated with ‘No Child Left Behind’ [21-23]. Thus, school-based PE classes as currently offered may not provide children and youth with sufficient PA opportunities.

Public policy has potential to increase children’s PA participation in schools. By enacting policy, governments can effectively and equitably create school environments that support PA with little effort on the part of children [24]. Evidence suggests that policies that require participation in, or that specify minimum time requirements for PE have been effective in increasing the amount of PE offered in schools [25-27], in increasing student attendance to PE class [28], PA levels [28,29], and physical fitness [30]. Furthermore, increased time spent in PE/PA does not compromise, and may even improve academic performance [31-35]. School-based PA policies may offer similar benefits as PE policies, although few studies exist [36].

To increase children’s opportunities for PA, several Canadian provinces and US states have adopted school-based daily PA (DPA) policies. These policies stipulate minimum PA time requirements for children and youth during, and in some cases also outside of, school hours. Schools and School Boards may enact their own policies provided they meet or exceed these minimum provincial/state standards. It is not clear why some jurisdictions have adopted DPA policies, while others have not. The impetus for policy change can originate within a polity, such as when interests groups coalesce around an issue and advocate for change [37]. Alternatively policy adoption can also be stimulated through processes of diffusion [38], in which governments learn from the experiences of other jurisdictions, rather than develop their own novel policies for each specific issue [37-39]. Policy diffusion is a common phenomenon. Just as diffusion of tobacco control legislation helped to de-normalize tobacco consumption [38], diffusion of school-based DPA policies has potential to inspire widespread normative change related to children’s PA opportunities. An understanding of DPA policy trajectories can inform efforts to stimulate and accelerate policy adoption among jurisdictions that have been resistant or slow to adopt such policies. Policy disparities can lead to differential child health outcomes, underscoring the need to examine the articulation of PA policies across Canada [38].

Policy adoption and diffusion are not discrete and bounded events, but rather context-sensitive processes that unfold over time. While previous investigations have explored cross-sectional predictors of PA-related policy adoption and diffusion [40-43], few have investigated the processes underlying these outcomes, and none to our knowledge have focused on DPA policies in schools. Furthermore, the level of implementation and impact of these policies within the Canadian context has not been synthesized. Thus, the purpose of this study was to understand the processes underlying adoption and diffusion of Canadian provincial/territorial DPA policies, and to review evidence regarding their implementation and impact.

## Methods

### Study design

To understand the dynamics surrounding adoption and diffusion of DPA policies, we adopted a multiple case history methodology in which we traced the chronological trajectory of DPA policies among Canadian provinces by compiling timelines detailing key historical events that preceded policy adoption [44]. Diffusion of Innovations theory provided an organizing conceptual framework for the analyses [45]. The theory posits that the key characteristics of adopters (i.e. their innovativeness, as reflected by their position in a diffusion curve), of the innovation, and key contextual factors such as communication channels and social networks are strong determinants of adoption [45]. Similarly, Shipan and Volden [39] propose that policy diffusion is conditioned on the nature of policies, characteristics and capacities of adopters, and contextual factors. Accordingly, the current analysis characterizes adopters based on their degree of innovativeness, describes the content of their DPA policies, and the context surrounding policy adoption. To complete the policy cycle, we performed a systematic review of studies that have evaluated adoption, diffusion, implementation or impact of school-based Canadian DPA policies.

### Data collection

#### Identification of adopters

First, websites of the Ministries of Education in each Canadian province/territory were searched to determine which jurisdictions had school-based DPA policies, and the time of policy adoption and implementation. We searched the Lexis Nexis Legislation database [46] and the Prevention Policies Directory [47] to identify DPA policies that may have been missed, or that had been proposed but not enacted. A jurisdiction was considered to have a DPA policy if it had statutory legislation (laws enacted by a provincial legislature) or administrative laws (rules and regulations developed by provincial ministries) specifying PA time requirements for school-aged children that were administered at the school level [26,48]. These jurisdictions are hereafter referred to as ‘adopters’. Jurisdictions without DPA policies, or with PE policies that did not specify minimum PA time requirements were designated as ‘non-adopters’. Within non-adopter provinces/territories there may have been smaller jurisdictions that had adopted DPA policies, however we limited our review to those that were enacted at the provincial level (i.e. province-wide).

#### Policy content and context

Once adopters were identified, government websites provided a starting point to understand the content of the various policies, the context within which they were developed and implemented, and to locate additional DPA-related documents, including guidelines, implementation plans, memoranda, policy frameworks and strategies, school curricula, action plans, newsletters, press releases, evaluations, and other documents related to the genesis and evolution of DPA policies over time. General web-based searches helped to locate additional documents. Once relevant sources were identified, we used a snowballing procedure to follow links within these documents to others that were of interest. Given the breadth of the search processes and terms, we did not attempt to record or screen all hits or the number of documents reviewed, and focused instead on retrieving and reviewing all documents that described polices or programs related to PA and/or healthy living initiatives in schools [44]. We requested 2 Alberta-based documents that had been cited, but were no longer available online from report authors.

This body of evidence was used to construct a comprehensive timeline for each adopter on which we plotted key policy cycle-related events (e.g. policy adoption and implementation) relative to key contextual events (e.g. major reports, activities of advocacy groups), spanning the earliest located reference to PA promotion in schools, to the present day (i.e. August, 2014). We contacted government officials to clarify the information contained within some documents, and to confirm the specific timing of adoption and implementation of all policies. No provincial ministries could confirm dates on which DPA policies were adopted; therefore, the public announcement date for each policy was used as the proxy date for policy adoption. In all cases, policies were publicly announced prior to their implementation. An independent reviewer subsequently reviewed all documents in each timeline to determine which documents to retain in the final analysis using a more targeted set of criteria. Specifically, documents were excluded that were unrelated to the role of schools in provision of PA/PE, had not been mentioned within other sources as having informed DPA policy-related activities, or for which there was no clear evidence of a contribution to placing PA in schools on the policy agenda. In addition, timelines were truncated according to the date of DPA policy adoption. A total of 59 documents were included in the final provincial timelines.

#### Policy evaluation

**Figure 1:**
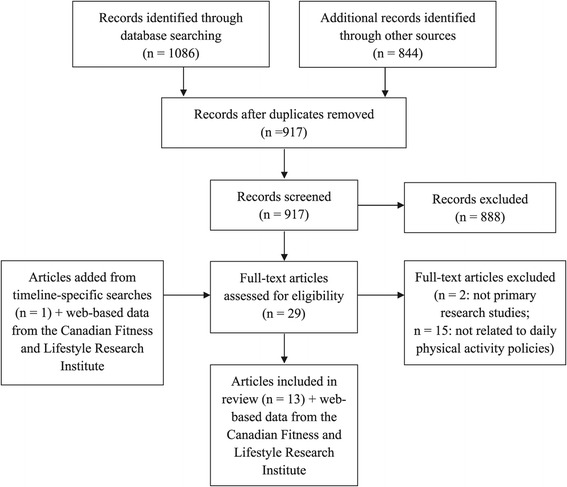
PRISMA flow diagram.

Next, a systematic search was performed to identify studies that had evaluated any aspect of school-based DPA policies. An information specialist designed and executed the search using a scoping review framework [49]. Electronic databases (Ovid Medline, Ovid PsycINFO, Ovid ERIC, and SPORTDiscus with Full Text via EBSCOhost) were searched using subject headings and text words for concepts related to policies, schools, and PA (Additional file 1). Database searches were limited to English and French references published between 2003 and August, 2014. A total of 1086 citations were identified through these searches, with an additional 844 identified by checking websites for known research centers (Rudd Center for Food Policy and Obesity, Bridging the Gap, and Active Living Research) and from reference lists from key systematic and literature reviews. Websites of groups known to be conducting, or to have conducted PA-related research in Canadian schools were also hand searched to identify additional DPA-related studies (Compass, SHAPES, Healthy Kids, Living School, People for Education, Active Schools! BC). These sources did not yield additional articles for the systematic review, although they did yield documents for the broader document review. Following removal of duplicate records, 917 unique citations remained (Figure 1).

A single reviewer examined all titles and/or abstracts to remove irrelevant studies. Articles were eligible for inclusion if they met the following criteria: 1) Original research study published in a peer-reviewed journal in English or French; and 2) Evaluated adoption, diffusion, implementation or impact of school-based DPA policies in Canada. The entire article was retrieved and screened if abstracts were not available or were not sufficiently detailed. Two reviewers, working independently, then examined the full text of the remaining articles (n = 29) for compliance with inclusion criteria. Disagreements were resolved through consensus discussion. Two papers were excluded because they were not primary research studies, while 15 were excluded because they did not concern Canadian DPA policies. Supplemental searches of reference lists of included papers and of related reviews and commentaries identified 1 additional article, along with web-based data from the Canadian Fitness and Lifestyle Research Institute describing objective measures of children’s PA levels by province. Although not published in a peer-reviewed journal, the latter data were included as they provided national, robust and objective measures of school-aged children’s PA levels by province/territory throughout the time frame during which DPA policies were adopted and implemented. Thus, the final review included 13 articles describing 9 unique studies, and data from the Canadian Fitness and Lifestyle Research Institute. References within these papers to additional documents describing the history of DPA-related legislation within each province were followed up and added to timelines as appropriate.

### Data analyses

#### Characterization of adopters

Rogers’ Diffusion of Innovations framework categorizes adopters into 1 of 5 adopter categories (innovators, early adopters, early majority, late majority, laggards) based on the time at which they adopt a new idea, which defines their position within a typical S-shaped adoption curve [45]. Given the small number of provinces with DPA policies, an adoption curve could not be empirically fitted. In addition, there is no consensus as to whether adopters should be classified on the basis of when they decide to use, or actually begin using a new idea [45]. Because the number of adopters was small we were able to use a hybrid approach in which we classified adopters into categories based on their earliness in *both* adopting and implementing DPA policies relative to other provinces over the 7 year time frame during which adoption occurred (2003–2010) .

#### Policy content

DPA policy statements for each province were reviewed to identify major elements of each policy including: type of language used (e.g. prescriptive, specific), number of minutes of PA required and over what time frame (e.g. sustained, smaller time segments), types of PA required (e.g. MVPA), time of day during which PA had to be performed (e.g. instructional time, non-instructional time), grade-levels included, implementation timelines and enforcement mechanisms. Policy strength was evaluated using the method of Carlson et al. [50] by 2 independent reviewers. Weak policies were those that were vague and used non-specific language to provide suggestions or recommendations, rather than requirements. Moderately strong policies used specific language to mandate minimum PA time requirements and MVPA (as opposed to PA, which is vague and can include light intensity PA). Strong policies were required to meet the criteria for moderately strong policies and to include mechanisms for implementation and monitoring.

#### Policy context

Documents associated with all timeline events were re-reviewed in detail and summarized in narrative format for each province, following a standard chronological outline that highlighted key contextual events. The study’s second author, who had been responsible for developing the initial comprehensive timelines, reviewed the final timelines and narratives for accuracy and completeness.

#### Policy evaluation

Data from included studies were extracted by 2 independent investigators, who examined study objectives, design, sampling and measures, results and conclusions. Disagreements were resolved through discussion. Presentation of results was limited to DPA-specific findings, as other findings found within some papers (e.g. individual predictors of PA behaviors) were beyond the scope of the current review.

## Results

### Characterization of adopters and policy content

**Figure 2:**
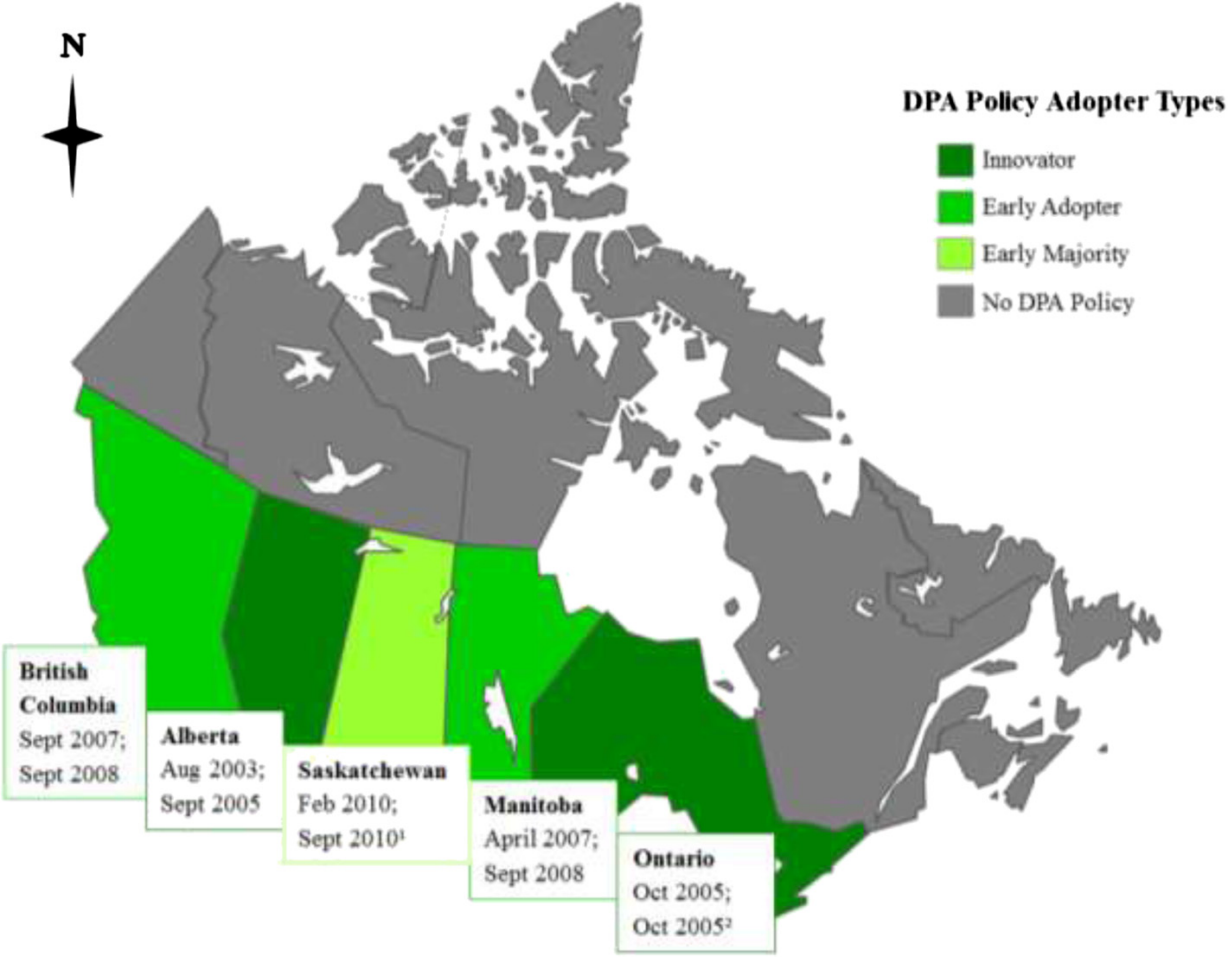
Map of time of adoption and implementation of Canadian daily physical activity policies. Provinces are listed in bold, followed by the date of DPA policy adoption and the date of DPA policy implementation. The date on which DPA policies were publicly announced was used as a proxy for the date of policy adoption. ^1^Optional implementation. ^2^Full implementation expected by the end of the 2005–06 school year. DPA: daily physical activity.

Five provinces of Canada’s 13 provinces and territories (38.5%) have province-wide DPA policies: British Columbia (BC), Alberta (AB), Saskatchewan (SK), Manitoba (MB) and Ontario (ON) (Figure 2), representing 72% of Canadian children aged 5–19 years. Table 1 designates each province within an adopter category and summarizes the content and strength of each province’s DPA policies. AB and ON were classified as innovators because they announced policies within a few years of each other (2003 and 2005, respectively) and implemented them in the same year (2005). MB and BC adopted and implemented policies virtually simultaneously (2007–08), but several years after AB and ON, marking them as early adopters. SK lagged behind the others by several years (2010), and was therefore classified as part of the early majority, although it is too early in the course of diffusion to know for certain whether this categorization is appropriate. If SK truly is part of the early majority, then diffusion should accelerate at some point in the near future. By contrast, if other provinces fail to adopt policies in a timely manner, or at all (in which case the S-shaped curve may not apply), then SK may be better characterized as an early adopter, or alternatively as the final adopter.

**Table 1:** **Summary of provincial daily physical activity policies**

**Province**	**Grade**	**Minutes and distribution**	**Type of PA**	**Delivery of PA** ^**1**^	**Strength** ^**2**^	**Date adopted** ^**3**^	**Date implemented**	**Key adoption factors**	**Monitoring of implementation and impact**
***Innovators 2003-2006***									
**AB** [56,66,158,159]	Grades 1-9	≥30 mins/d; may be offered in smaller time segments	PA should vary in form and intensity	Activities organized by the school; can include instructional or non-instructional hours (school-based)	Weak	Aug, 2003	Sept, 2005	Convergence of Kingdon’s 3 policy streams: problem, solution, and policy; strongly influenced by the actions of the Minister of Learning	School authorities responsible to monitor implementation; DPA survey of educators conducted by AB Education
**ON** [17,68,70,71]	Grades 1-8	≥20 mins/d; sustained	Sustained MVPA	During instructional hours	Moderate	Oct, 2005	Oct, 2005, with full implementation by end of 2005/06 school year	DPA in schools was part of the 1998 Health and PE curriculum, the Active2010 sport and PA strategy and Living School, and was supported by the Chief Medical Officer of Health	School Boards responsible to monitor DPA implementation
***Early adopters, 2006-2010***									
**MB** [75,79,80,160,161]	Grades 11-12	1 PE/Health Education credit required per grade including PA practicum of ≥ 55 hrs	PA practicum focusses on MVPA + ≥ 1 of: strength, endurance, flexibility	In-, out- or a combination of in- and out-of-class/school time with adult sign off for out-of-class/school PA	Moderate	Apr, 2007	Sept, 2008	The Healthy Kids, Healthy Futures Task Force Report recommended mandating PE for grades 11-12	Students must complete a personal fitness portfolio; teachers document student-level completion on report cards
**BC** [89,91,119,137,162,163]	Kinder-garten	15 mins/d (half-day)30 mins/d (full day); may be offered in smaller time segments of ≥ 10 mins	Includes endurance, strength and/or flexibility activities	Part of students’ educational program; can include instructional or non-instructional hours (school-based)	Weak	Sept, 2007	Sept, 2008	Action Schools! BC was an early model of DPA that proved efficacious and was disseminated across BC	School Boards develop their own policies and procedures to track DPA implementation; teachers document student-level achievement on term and final report cards
	Grades 1-7	30 mins/d; may be offered in smaller time segments of ≥ 10 mins	Includes endurance, strength and/or flexibility activities	Part of students’ educational program; can include instructional or non-instructional hours (school-based)	Weak	Sept, 2007	Sept, 2008	Action Schools! BC was an early model of DPA that proved efficacious and was disseminated across BC	School Boards develop their own policies and procedures to track DPA implementation; teachers document student-level achievement on term and final report cards
	Grades 8-9	30 mins/d; may be offered in smaller time segments of ≥ 10 mins OR 150 mins/wk	Includes endurance, strength and/or flexibility activities OR MVPA	Part of students’ educational program; can include instructional or non-instructional hours (school-based) OR In- or out- of school PA documented by student	Weak	Sept, 2007	Sept, 2008; as of Sept, 2011 schools select 30 mins/d DPA OR 150 mins MVPA/wk	Action Schools! BC was an early model of DPA that proved efficacious and was disseminated across BC	School Boards develop their own policies and procedures to track DPA implementation; teachers document student-level achievement on term and final report cards
	Grades 10-12	150 mins/wk	MVPA	In- or out- of school PA documented by student	Moderate	Sept, 2007	Sept, 2008	Action Schools! BC was an early model of DPA that proved efficacious and was disseminated across BC	School Boards develop their own policies and procedures to track DPA implementation; teachers document student-level achievement on term and final report cards; graduation requirement
***Early majority, 2010 - 2014***									
**SK** [100-103,105]	All students	30 mins/d	MVPA	Not specified; School Boards expected to develop new or strengthen existing PA policies consistent with general government guidelines	Weak	Feb, 2010	Sept, 2010 (optional)	The SK population health strategy, SK *in motion*, and implementation of Quality Daily PE focused attention on the need for greater PA in schools	School Boards responsible to ensure that policy results in increased PA for all children

DPA: Daily physical activity; MVPA: moderate-to-vigorous physical activity; PA: physical activity; PE: physical education.

^1^Instructional and in-class time both refer to teacher-initiated and supervised activities that take place in a formal classroom setting. Non-instructional and out-of-class time can include school and non-school-based activities [79].

^2^Policy strength was evaluated using the method of Carlson et al. [50] by 2 independent reviewers.

^3^The date on which DPA policies were publicly announced was used as a proxy for the date of policy adoption.

Although the underlying objectives of the 5 policies are similar, namely to alter the school environment in order to increase children’s PA levels and improve their health, there are clear differences among them. The ON policy, for instance, requires ≥ 20 minutes of sustained daily MVPA, whereas others do not require sustained activity and AB does not require MVPA at all. ON is also distinct in requiring ≥ 20 minutes of DPA, whereas all other provinces mandate ≥ 30 minutes of PA daily or ≥ 150 minutes weekly. The requirement for DPA within MB’s new PE/Health Education curriculum applies exclusively to students in grades 11–12, whereas the AB and ON policies apply only to younger students (grades 1–9 and 1–8, respectively). BC and SK are the only provinces with DPA policies that apply to students in all grades. The policies also differ as to when DPA must be provided. In ON, DPA must be delivered during instructional time, whereas AB allows DPA to be provided at any point during the school day. Provinces that mandate DPA in higher grades permit some or all PA to be achieved outside of school hours, with documentation by students or responsible adults. All policies allow PE time to be used to meet policy objectives. The SK policy is distinct in that it contains few specific provisions and its implementation was characterized as optional during discussions (Government of Saskatchewan, personal communication, September 4, 2014).

### Policy context

Table 2 summarizes key contextual events preceding adoption and implementation of DPA policies in each province.

**Table 2:** **Policy timelines by province**

**Date [reference]**	**Event**
**Alberta**	
1984 [18,52]	The Health and Physical Education Council of the AB Teachers’ Association releases 2 position papers calling for 30 minutes of Quality Daily PE in AB schools.
1989 [9]	The Health and Physical Education Council of the AB Teachers’ Association develops Schools Come Alive to increase students’ awareness and skills for active living.
1990 [54]	The AB Coalition for Healthy School Communities is created to facilitate networking and information sharing among those with an interest in comprehensive school health.
1995 [53]	Schools Come Alive releases a strategic plan to make PA and PE priorities in AB schools.
1998 [63]	AB’s Active Living Strategy recommends that AB schools create opportunities for students to be physically active each school day.
2000 [64]	AB releases a new PE curriculum emphasizing PA and attainment of life-long active living.
2001 [53]	Schools Come Alive creates Ever Active Schools as a pilot project to encourage active living initiatives in schools.
2001 [57]	The Coalition for Active Living reports PA has declined in Canada partly because PE has been cut in schools.
2001 [60]	The Mazankowski report suggests students should have the opportunity for regular exercise as part of every school day.
2002 [61]	Delegates at the AB Future Summit propose re-introducing daily PA into the school curriculum.
2003 [62]	AB’s Commission on Learning recommends a new wellness program for students from kindergarten to grade 12 that includes some form of daily activity.
2003 [56,65]	AB Learning announces a daily PE policy for students in grades 1–12 (later corrected to DPA).
2005 [66,67]	AB implements a DPA policy for grades 1–9. Plans to implement DPA in high schools are cancelled.
**Ontario**	
1998 [17]	ON releases a Health and PE curriculum requiring student participation in daily, sustained, moderate or vigorous PA (with minimum time expectations for some grades).
2001-02 [69]	ON develops a Stakeholder Sport Action Plan to support the Canadian Sport Policy^1^.
2002 [73]	The Ministry of Health and Long-Term Care proposes a school-based, province-wide initiative for primary prevention of diabetes.
2004 [70,71]	ON implements a Healthy Schools Program.
2004 [73]	A comprehensive school health initiative called Living School is launched, and includes DPA.
2004 [72]	The Chief Medical Officer of Health releases a report recommending policies be developed to support the ACTIVE2010 Sport and Physical Activity Strategy, and that quality daily PE and PA opportunities be provided in schools.
2004 [69]	ON’s ACTIVE2010 Sport and Physical Activity Strategy supports implementation of 20 minutes of DPA in elementary schools.
2005 [68]	ON announces a DPA policy for grades 1–8.
2006 [68]	Full implementation of the DPA policy is expected by the end of the 2005–06 school year.
**British Columbia**	
1983 [86]	The Directorate of Agencies for School Health (DASH) BC is established and later introduces the concept of comprehensive school health in BC schools.
1989 [86]	A Government Office of Health Promotion is established in BC.
1992 [86]	A Healthy Schools program is launched throughout BC.
2002 [86]	The Healthy Schools program ends.
2001-02 [88,89]	Stakeholder consultations to identify the strategic agenda for action on PA in BC schools leads to development of Action Schools! BC.
2003 [86,87]	The BC Provincial Health Officer’s report recommends a re-commitment to support Healthy Schools initiatives.
2003-04 [89-91]	Action Schools! BC is evaluated and proves acceptable, feasible and efficacious.
2003-04 [93]	BC develops a chronic disease prevention strategy, Healthy BC 2010.
2004 [92]	Widespread dissemination of Action Schools! BC is funded through the Healthy Schools Program.
2005 [93,94]	The BC Healthy Living Alliance circulates The Winning Legacy to each Ministry to advocate for multi-level interventions (including school-based initiatives) to curb chronic disease.
2005 [93]	Healthy BC 2010 is renamed ActNowBC and aims to make BC a North American leader in healthy living and physical fitness.
2006 [95]	The BC Medical Association recommends 30 minutes of DPA in schools to the BC Select Standing Committee on Health.
2006 [96]	The Select Standing Committee on Health recommends that every student be required to participate in DPA and that Action Schools! BC be expanded.
2007 [97]	The BC government announces that DPA will be mandated in all BC schools (kindergarten to grade 12).
2008 [97]	A DPA policy is implemented in all BC schools.
**Manitoba**	
1975 [74]	A MB Physical Education Working Group proposes that all MB schools be required to offer 40 minutes of PE per day, including 20 minutes of vigorous PA.
2000 [75]	MB adopts an integrated approach to PE/Health Education programming that recognizes the value of regular PA.
2000 [76]	The Healthy Child MB Strategy is implemented that focusses on creating child-centered public policy.
2000 [77]	Nurses-in-Schools is introduced to support public health in schools.
2002 [78]	The MB Physical Activity Action Plan recommends mandating daily PE from kindergarten to grade 12.
2003 [77]	Nurses-in-Schools expands to become MB Healthy Schools, a program that draws on the principles of comprehensive school health.
2005 [80]	The Healthy Kids, Healthy Futures Task Force Report recommends changes to the MB PE/Health Education curriculum and a voluntary *in motion* program to engage students in 30 minutes of DPA.
2005 [79]	The MB government pledges to implement all 47 of the Task Force’s recommendations.
2005 [82,83]	MB *in motion* is launched to increase PA in MB.
2005 [84]	Healthy Schools and MB *in motion* partner to offer Healthy Schools *in motion* to support 30 minutes of DPA for all students.
2007 [79,85]	The MB government mandates the amount of time students in kindergarten to grade 10 must spend in PE/Health Education classes.
2008 [79]	The MB government implements a PE/Health Education curriculum for students in grades 11–12. Students in grades 11–12 are required to complete 2 PE/Health Education credits for graduation, including ≥ 55 hours of MVPA per credit.
**Saskatchewan**	
2001 [98]	School^PLUS^ is released, outlining a vision for schools to meet the needs of the ‘whole’ child.
2001 [99]	In response to the Clear Lake Accord^1^, SK releases a provincial strategy with a goal of ensuring schools provide DPA called A Physically Active SK.
2003 [101]	SK *in motion* is launched to increase PA across the province.
2004 [100]	The SK population health strategy outlines a plan to support regular PA in schools.
2006 [101,102]	SK *in motion* changes its focus to school-aged children and promotes 30 minutes of PA at home, 30 minutes at school and 30 minutes in the community. *In motion* schools provide ≥ 30 minutes of DPA.
2009 [103,104]	Quality Daily PE is reported to be widely implemented in SK.
2010 [105]	A provincial DPA policy is announced for all schools.
2010	Voluntary^2^ implementation of the DPA policy begins.

DPA: Daily physical activity; MVPA: moderate-to-vigorous physical activity; PA: physical activity; PE: physical education.

^1^Provincial response to a federal policy.

^2^Government of Saskatchewan, personal communication, September 4, 2014.

#### Innovators, 2003–2006

##### Emergence of DPA policies in AB

Established in 1962, the Health and Physical Education Council of the AB Teachers’ Association provided early leadership for increased provision of PE/PA within schools [51]. In 1984, the Council released 2 position papers calling for 30 minutes of Quality Daily PE in AB schools [18,52], while in 1989 it developed Schools Come Alive, a pilot project to increase students’ awareness and skills for active living [9]. The program was subsequently expanded to a provincial level and aimed to integrate active living initiatives within AB schools [53]. Among the program’s most notable initiatives were a 1995 strategic plan for prioritizing school-based PA and PE, and creation of Ever Active Schools in 2001, which later became an important provider of DPA-related resources [53]. The AB Coalition for Healthy School Communities, a network of individuals and organizations committed to school heath, was another early player in efforts to embed health promotion within AB schools [54].

Although these early initiatives were important, according to a retrospective qualitative analysis of the adoption of DPA policies in AB, DPA was specifically mandated in Alberta schools because in 2003 the 3 streams within Kingdon’s streams model [55] (i.e. problem, solution and politics) converged [56]. First, a number of high profile reports focused attention on the contribution of inadequate PA to childhood obesity and poor health outcomes (i.e. problem stream) [57-59]. Second, school-based DPA was acknowledged in a number of influential venues as a potentially viable and effective means to increase children’s PA levels (i.e. solution stream) [18,60-63]. AB’s 1998 Active Living Strategy was among these reports which recommended that schools create opportunities for students to be physically active each school day [63]. The emphasis on PA and attainment of life-long active living within AB’s PE curriculum at the time [62,64] and creation of Ever Active Schools [53] also helped to build momentum for, and establish a supportive context for school-based DPA. In 2003, Kingdon’s 3 streams were linked when the Minister of Learning used his ministerial power to propose a DPA policy as a viable political solution to the problems of inactivity and obesity among children (i.e. politics stream) [56]. Although initially announced as a daily PE policy for grades 1–12 [65] this error by the Minister was quickly corrected and a DPA policy was implemented in September, 2005 in grades 1–9 [66]. Plans to implement DPA in high schools in September, 2006 were cancelled, reportedly due to stakeholder concerns [67].

##### Emergence of DPA policies in ON

In October, 2005, ON became the second Canadian jurisdiction to announce a DPA policy for schools [68], although as early as 1998 the ON Health and PE curriculum required student participation in daily, sustained, moderate or vigorous PA (with time expectations for some grades) [17]. The new policy, which was to be implemented by the end of the 2005–06 school year [68], was occasioned by the confluence of 3 high-profile reports. First, in response to development of a Canadian Sport Policy in 2000–01, ON developed a Sport Action Plan Framework and subsequently a full ACTIVE2010 Sport and Physical Activity Strategy in 2004 [69]. The strategy represented a critical juncture, as in it the Ministry of Health Promotion highlighted the importance of policy in creating change and pledged to support implementation of 20 minutes of DPA in elementary schools [69]. The strategy also led to development of the ON Healthy Schools Program in 2004, of which DPA policies were to become a part [70,71].

Also in 2004, the ON Chief Medical Officer of Health released a report entitled “Healthy Weights, Healthy Lives” that recommended policies be developed to support the ACTIVE2010 Sport and Physical Activity Strategy, and that quality daily PE and PA opportunities be provided in ON schools [72]. Finally, in response to a 2002 report by the Ministry of Health and Long-Term Care, funding was allocated to develop a provincial school-based initiative for diabetes prevention, which became known as Living School [73]. Based upon the tenets of comprehensive school health (synonymous with Health Promoting Schools in Europe and Coordinated School Health in the US), the program involved implementation of DPA, and provided an early model of the potential of DPA to increase children’s PA levels [73].

#### Early adopters, 2006–2010

##### Emergence of DPA policies in MB

In 1975, a MB Physical Education Working Group proposed that all MB schools be required to offer an average of 40 minutes of PE per day, including 20 minutes of vigorous PA [74]. Subsequent to this early recommendation, the genesis of MB’s DPA-related policies appears to have been in the year 2000, through several key activities. First, that year MB adopted an integrated approach to PE/Health Education programming [75]. A key principle was that students should be physically active on a regular basis as part of the learning process [75]. Also in 2000, the government of MB implemented the Healthy Child MB Strategy that focused on creating child-centered public policy [76]. That same year, “Nurses-in-Schools” was introduced to support public health in schools [77], and, following a provincial consultation in 2002, expanded to become Healthy Schools, a program that draws on the principles of comprehensive school health [77]. Another key event in 2000–02 was the development of the MB Physical Activity Action Plan which recommended mandating daily PE from kindergarten to grade 12 [78].

Although these early activities built momentum for PA/PE promotion in MB schools, the precipitating event for the emergence of DPA policies in MB occurred in 2005, with the release of the Healthy Kids, Healthy Futures Task Force Report [79]. Announced in August, 2004, the Task Force heard testimony recommending that PE be mandated from kindergarten to grade 12, while others supported provision of DPA [80,81]. In its final report, the committee recommended that the government mandate the amount of time students in kindergarten to grade 10 spent in PE/Health Education classes, develop a PE/Health Education curriculum for grades 11 and 12, and require students in grades 11 and 12 to take 2 semesters of the course for graduation [80]. The Task Force also recommended that the provincial government introduce a voluntary MB *in motion* program in which students from *in motion* schools must participate in 30 minutes of DPA [80].

The MB government pledged to implement all 47 of the Task Force’s recommendations [79], and shortly thereafter launched MB *in motion* to increase PA in MB by 10% by 2010 [82,83]. Healthy Schools and MB *in motion* subsequently partnered to offer Healthy Schools *in motion. In motion* schools commit to working towards the goal of providing 30 minutes of DPA for all students through any combination of PE, PA breaks and programs, intramurals and special events [84]. The other PE/Health Education-related Task Force recommendations were fulfilled in 2007–08, including requiring students in grades 11 and 12 to complete 2 PE/Health Education credits for graduation, and although it was not a Task Force recommendation, students in grades 11 and 12 were additionally required to participate in 55 hours of MVPA per PE/Health Education course [79,85]. Because only the grade 11–12 PE/Health Education curricula requires participation in a minimum amount of PA, it alone was considered a DPA policy for the purposes of the current analysis.

##### Emergence of DPA policies in BC

Established in 1983 by the BC Ministries of Education and Health in partnership with community agencies, the Directorate of Agencies for School Health BC first introduced the concept of comprehensive school health in BC schools [86]. Later, a government Office of Health Promotion was established, launching a Healthy Schools program throughout the province in 1992 [86]. Although widely disseminated, the program was subsequently ended in 2002 [86]. In 2003, the comprehensive school health movement regained momentum following release of the Provincial Health Officer’s Report which recommended a re-commitment to support Healthy Schools initiatives [86,87].

It was in this context that in 2001–02, the BC ministries of Health and Tourism, Sport and the Arts initiated stakeholder consultations to identify the strategic agenda for action on PA in BC, with a focus on schools [88]. A proposal to mandate daily PE from kindergarten to grade 12 was opposed by education stakeholders, and instead, a flexible ‘active school’ model for PA promotion in elementary schools emerged [88,89]. The program, known as Action Schools! BC, provided children with small PA breaks throughout the school day (in addition to scheduled PE classes), with the goal of providing 150 minutes/week of moderate intensity PA [89]. A 2003–04 evaluation supported the program’s acceptability, feasibility and efficacy in increasing children’s opportunities to be physically active, PA participation, and in improving their fitness and health-related outcomes [89-91]. Therefore, widespread dissemination of Action Schools! BC was funded through the Healthy Schools Program [92].

The impact of Action Schools! BC was widespread in terms of enhancing political support for promoting school-based PA in BC [88]. Similarly, the Healthy Schools Program helped to embed school health as a core element within the business plans of key government ministries [92]. The province’s 2003–04 chronic disease prevention strategy, Healthy BC 2010, later renamed ActNowBC, was also influential, as its focus on making BC a North American leader in healthy living and physical fitness helped to create a climate in which PA was supported and promoted across multiple government departments and societal sectors [93]. Advocacy by the BC Healthy Living Alliance for multi-level interventions (including school-based actions) to address chronic disease was also influential in this respect [93,94]. Ultimately, these capacity building and awareness raising activities culminated in a recommendation by the BC Medical Association to BC’s Select Standing Committee on Health to implement 30 minutes of DPA in schools [95], and a subsequent strong endorsement by the committee that every student in BC’s educational system be required to participate in DPA, and that Action Schools! BC be expanded even further [96]. In 2007, the BC government announced that DPA would be mandated in all BC schools in the 2008 school year, as a complement to, rather than a replacement for Actions Schools! BC [97].

#### Early majority, 2010–2014

##### Emergence of DPA policies in SK

Discussion of the role of schools in society and in meeting the holistic needs of children surfaced in a number of forums in SK in the 1990’s [98]. In response, a Task Force was formed that in 2001, produced a report outlining a vision to expand the role of schools beyond education to meet the needs of the ‘whole’ child which became known as School^PLUS^ [98]. That same year, the SK government released ‘A Physically Active SK’ provincial strategy, with a goal of ensuring schools involved students in DPA [99]. The strategy represented the fulfillment of SK’s pledge, along with the other federal-provincial/territorial ministers responsible for fitness, active living, recreation and sport to reduce physical inactivity among Canadians by 10% by 2003 [99]. As a complement to this strategy, a SK population health strategy was developed in 2004, outlining a government plan to support PA through increasing opportunities for, and reducing barriers to regular PA in schools [100]. Suggested actions included promoting PA policies in school divisions [100].

In 2003, SK *in motion* was launched to increase PA across the province, but in 2006 switched focus to school-aged children and youth, with a goal to ‘get kids moving’ through 30 minutes of PA at home, 30 minutes at school, and 30 minutes in the community [101]. *In motion* schools commit to providing ≥ 30 minutes of DPA for all students [102]. SK *in motion*, along with Quality Daily PE [103] were widely implemented across the province [104] prior to the announcement of a provincial DPA policy in 2010 [105]. School divisions began optional implementation of DPA in September, 2010 (Government of Saskatchewan, personal communication, September 4, 2014).

#### Non-adopters

##### Quebec, the Atlantic provinces (Nova Scotia, New Brunswick, Prince Edward Island, Newfoundland and Labrador), and the northern territories (Northwest Territories, Yukon, Nunavut)

Non-adopters consist of provinces/territories that have not yet adopted a DPA policy, although some may never adopt a policy. Currently, non-adopters are co-located geographically in Canada’s eastern provinces and northern territories, and with the exception of Quebec, are among the least affluent and least populous regions of the nation [106,107]. Notably, however, 6 of these 8 provinces/territories have comprehensive school health initiatives in place or underway, which suggests that attention to school environments is a priority [108]. Action to address PA in schools is also evident in all of these jurisdictions. For example, in 2006 a bill in New Brunswick proposed mandating 150 minutes/week of PA for all students [109], while in Nova Scotia, a 2007 bill proposed that students in grades 1–9 receive at least 30 minutes of DPA [110]. Neither of these bills was passed. Quebec en Forme is an example of another major provincial initiative aimed at increasing PA levels among children and youth [111].

#### Canada

Although we focused on describing provincial-level policy influences, at least two developments at the national level have been foundational for development of DPA policies in Canada, and are therefore briefly mentioned here. In 1974, the School Physical Activity Program Special Interest Group introduced the concept of Quality Daily PE within Canada [15], and in 1983, a proposal for a Quality Daily PE program emerged [15,112]. Officially launched in 1988, the program provides minimum standards for PE programs, including ≥ 30 minutes of daily PE with a high degree of student participation [112]. The Quality Daily PE concept, including provision of DPA, was immediately and strongly endorsed by the federal Minister of State, Fitness and Amateur Sport at the time [15]. Formation of the Pan-Canadian Joint Consortium for School Health in 2005 marked another key turning point for school health in Canada [113]. A partnership of federal, provincial, and territorial governments, the Consortium aims to build capacity to promote school health and to act as a catalyst for collaborative activities and actions [113]. Importantly, the Consortium has provided a venue to share information regarding DPA policies and programs across Canada [92].

### Policy evaluation: Findings from the systematic review

#### Adoption and diffusion

One study examined adoption of DPA policies in AB (Table 3). No studies examined diffusion of DPA policies.

**Table 3:** **Summary of study findings**

**Study**	**Type of evaluation**	**Study design**	**Time frame**	**Population and setting**	**Measures**	**Outcome variables**	**Results**
**Alberta**							
**Gladwin et al., 2008** [56]	Adoption	Qualitative	Not stated	20 purposively selected key informants involved in school-based PA policy processes; review of policy documents and websites.	Semi-structured interviews and document reviews.	Policy processes that resulted in adding DPA, but not active transportation initiatives to the school curriculum.	DPA succeeded because Kingdon’s 3 streams (problem, solution, politics) converged, largely through the actions of the Minister of Learning who used his ministerial power to link the solution with the political stream.
**ONTARIO**							
**1) Faulkner et al., 2014** [133]**; 2) Stone et al., 2012** [136]	Implementation and impact	Cross-sectional	Apr-Jun, 2010; Sept-Dec, 2010; Apr-Jun, 2011	865 grade 5–6 students from 18 elementary schools in Toronto, ON. All schools invited to participate, 18 schools selected from among interested schools based upon neighborhood type and SES.	Students completed a survey, wore accelerometers for 7 days and had height and weight measured. Parents completed a survey and travel diary for their children. Principals completed a school health environment survey. Teachers provided classroom schedules to identify DPA and PE times.	1) Time spent in light-to-vigorous PA. Whether schools were in the initiation, action or maintenance stage of DPA. 2) Total PA counts and MVPA mins on school days and during school hours.	1) 11.1% of schools were in the initiation phase of DPA, 88.9% were in the action phase and none were in the maintenance phase. Students were not more physically active in schools that were in the action phase. 2) 49% of students were provided DPA every school day. Frequency of DPA positively associated with total PA and MVPA mins/d on weekdays and during the school day. Students who participated in DPA 5 d/wk had higher total counts and intensity of PA, and time spent in MVPA on school days and during the school day. PA bouts averaged 7.1 mins and none were ≥ 20 mins. Those who accumulated ≥ 1 bout of MVPA were more active and fewer were overweight and obese.
**1) Leatherdale et al., 2013** [145]**; 2) Leatherdale et al., 2014** [147]	Impact	Cross-sectional	2007-08	Convenience sample of 2326 grade 1–4 students/parents from 30 elementary schools in ON. Schools were purposively selected within public and separate school boards located in major geographic regions.	Students/parents completed a survey and students’ height and weight were measured. Administrators completed a school health environment survey.	Student activity levels (low, moderate, high) and BMI (normal, overweight, obese). Whether schools were in the initiation, action or maintenance stage of DPA.	1) DPA implementation was not associated with the odds of being overweight or obese. 2) DPA implementation was not associated with the odds of being highly or moderately active.
**1) Leatherdale et al., 2010** [134]**; 2) Hobin et al., 2010** [146]**; 3) Leatherdale, 2010** [165]	Implementation and impact	Cross-sectional	2007-08	Convenience sample of 2379 (studies 1–2) or 1264 (study 3) grade 5–8 students from 30 schools in ON. Schools were purposively selected within public and separate school boards located in major geographic regions.	Students completed a survey. Administrators completed a school health environment survey.	Student activity levels (low, moderate, high) and BMI (normal, overweight, obese). Whether schools were in the initiation, action or maintenance stage of DPA. DPA implementation models.	1) 0% of schools were in the initiation phase of DPA, 80% were in the action phase and 20% were in the maintenance phase. Implementation of DPA was not associated with the odds of being moderately or highly active. 2) DPA implementation models were: 70% offered DPA only on days without PE class, 20% offered DPA in addition to daily PE class, 10% offered DPA as part of daily PE class. DPA implementation models were not associated with the odds of being moderately or highly active. 3) DPA implementation was not associated with the odds of being overweight.
**Patton, 2012** [135]	Implementation	Cross-sectional	Not stated	145 teachers within 37 randomly selected schools in the Thames Valley District School Board, ON.	Teachers completed a survey.	Implementation and perspectives of DPA.	15.6% always conducted DPA on days when PE was not scheduled, 50.7% said there was not adequate time to conduct DPA, 60.9% said DPA should be integrated within the curriculum, 64.6% reported that administrators rarely or never monitored DPA.
**Robertson-Wilson and Levesque, 2009** [166]	Implementation	Qualitative	2005-07	Publicly available policy and other documents related to DPA implementation.	Document reviews.	Whether DPA implementation strategies fit Hogwood and Gunn’s 10 preconditions for perfect implementation.	Several preconditions (e.g. allocation of resources, task specification) have been considered, whereas others (e.g. sustainability of resources, evaluation plans, extent to which policy is valued) require additional attention to ensure optimal DPA implementation.
**British Columbia**							
**Watts et al., 2014** [137]	Implementation and impact	Cross-sectional, pre-post design	2007-08 and 2011–12 school years	Administrator surveys in all districts that consented. 2007–08: 384 elementary and 118 middle/high schools; 2011–12: 351 elementary and 125 middle/high schools.	Administrators completed a survey on PA practices at their school for grades 6, 8 and 10 students.	Mins and delivery format of PE, stakeholder support and level of implementation of DPA policies.	Implementation of DPA policies was 65%, 56% and 51% for grades 6, 8 and 10. Schools had higher odds of providing ≥ 150 mins PE/wk and provided more mins of PE to grade 8 and 10 students in 2011–12. Schools had higher odds of providing PA linearly to grade 8 and in a semester format to grade 10 students in 2011–12. Staff and parental support for PA policies increased in all schools, student support declined in elementary and increased in middle/high schools, principal support was unchanged.
**Masse et al., 2013** [119]	Implementation	Qualitative	2010-11 school year	Principals and teacher/school informants (n = 50) from a variety of school types and settings (n = 17 schools).	Semi-structured interviews.	Factors that impeded or facilitated implementation of DPA policies.	Perceived implementation ranged from 14%-90%. Schools implemented DPA policies through prescriptive and non-prescriptive approaches. DPA policies provided an advantage relative to the status quo, were compatible with school philosophies, and provided observable benefits. It was difficult to understand DPA policies, to fit them into already full schedules and policies increased teacher workload. Availability of resources and support were key facilitators.
**Manitoba**							
**Hobin et al., 2014** [148]	Impact	Longitudinal	Baseline measures were conducted in 2008 with annual follow-up to 2011 or completion of grade 12.	Convenience sample of grades 9–10 PE classes within 31 randomly selected secondary schools (n = 447 students) across MB.	Students completed a survey at baseline and wore accelerometers for 7 days once a year.	MVPA mins/d overall, and in students from schools in rural/urban and low/high SES areas.	The MVPA trajectories of adolescents declined 11.3%/yr from baseline to the last measurement. Students with low or moderate baseline MVPA and those attending schools in low SES and rural areas had slower rates of decline in MVPA.

DPA: daily physical activity; MVPA: moderate-to-vigorous physical activity; PA: physical activity; PE: physical education; SES: socioeconomic status.

#### Implementation

A total of 8 papers (6 studies) evaluated DPA policy implementation in ON and BC, including adherence to DPA policies, implementation models, processes and perspectives of implementation (Table 3). All of the studies were cross-sectional, although 1 used a pre-post comparison design. One study reported findings from a document review, another conducted key informant interviews, while the remainder used self-report methods (surveys and teacher logs) to assess implementation-related outcomes. None of the studies included control groups. Their collective results suggest moderate, but inconsistent implementation of DPA policies, however findings are tentative as there was significant variability in the methods used, and in the outcomes considered across studies. Moreover, there were very few studies, and samples sizes were small in all but 1 study. Process evaluations showed that an important barrier to implementing DPA policies was a lack of time to provide additional PA given competing curricular demands and priorities.

#### Impact

Impact of the ON, MB and BC DPA policies was assessed in 9 papers (5 studies) (Table 3). Seven papers were based on cross-sectional assessments, 1 used a pre-post comparison design, and 1 followed students longitudinally for up to 4 years. Three papers used accelerometry, while the remainder relied on self-reported surveys to assess PE delivery, PA behaviors, and BMI. While the number of students included in each study was large, the number of schools was small in all but 1 study, and none of the studies included control groups of students who were not exposed to DPA policies (i.e. from other jurisdictions). In addition, the Canadian Fitness and Lifestyle Research Institute used pedometers to quantify the number of steps taken daily by school-aged children on a national level [114,115]. Findings showed that with the exception of SK, in which a decline was observed, the average number of daily steps taken by school-aged children in provinces with DPA policies did not change between 2005 and 2011, and none differed from the national average (Table 4). Taken together, the available data suggest that Canadian DPA policies have had little to no impact on school-aged children’s PA levels or BMI; however findings are tentative in light of variability in methods used, outcomes considered, and the small number of studies.

**Table 4:** **Summary of findings from the Canadian Fitness and Lifestyle Research Institute’s Canadian Physical Activity Levels Among Youth (CANPLAY) studies** [114]

**Province**	**Year**	**DPA policy?**	**Approximate mean # steps taken**	**Comparison with other provinces/territories**	**Change compared to 2005–07 and 2007-09**
NU^1^	2009-11	No	>13,000	Not reported	Not reported
YK	2009-11	No	12,300^2^	More steps than NF, NS, NB	No change
NWT	2009-11	No	12,200^2^	More steps than NF	No change
BC	2009-11	Implemented in 2008	12,100^2^	More steps than NF, PEI, NS, NB Fewer steps than NU	No change
MB	2009-11	Implemented in 2008	12,100^2^	More steps than NF, PEI, NS, NB	No change
ON	2009-11	Implemented in 2005	11,700^2^	More steps than NF Fewer steps than NU	No change
Canadian average	2009-11	No national policy	11,600	n/a	Not reported
AB	2009-11	Implemented in 2005	11,500^2^	Fewer steps than NU	No change
SK	2009-11	Implemented in 2010	11,500^2^	Fewer steps than NU	Significant decline compared to 2005-07
QC	2009-11	No	11,400^2^	Fewer steps than NU	No change
NB	2009-11	No	11,200^2^	Fewer steps than MB, BC, YK, NU	No change
PEI	2009-11	No	11,200^2^	Fewer steps than MB, BC, NU	No change
NS	2009-11	No	11,100^2^	Fewer steps than MB, BC, YK, NU	No change
NF	2009-11	No	10,800^3^	Fewer steps than ON, MB, BC, YK, NWT, NU	No change

CANPLAY assessed the mean number of steps taken daily by Canadian children aged 5–19. Children wore the pedometer for 7 consecutive days [115]. Approximately 20,000 children were randomly selected and recruited in 2009–11 [115].

Provinces are listed in order from most to least steps taken.

AB: Alberta; BC: British Columbia; MB: Manitoba; SK: Saskatchewan; NB: New Brunswick; NF: Newfoundland and Labrador; NWT: Northwest Territories; NS: Nova Scotia; NU: Nunavut; PEI: Prince Edward Island; QC: Quebec; YK: Yukon.

^1^Data in Nunavut were collected using a different methodology [114,115].

^2^Not significantly different from the Canadian average of 11,600 steps per day [114].

^3^Significantly lower than the Canadian average of 11,600 steps per day (p < 0.05) [114].

## Discussion

Canada has enacted relatively little legislation designed to increase population-level PA. DPA policies represent an innovative approach to increase children’s PA in schools because they are intended to extend beyond PE classes to promote PA throughout the school day. Between 2003 and 2014, 5 of Canada’s 13 provinces and territories adopted school-based DPA policies, however our analysis suggests the strength of these policies was low to moderate. To explore factors associated with policy adoption and diffusion, in the following sections we compare and contrast the characteristics of adopters, the nature of their DPA policies, and the context surrounding DPA policy adoption. We conclude by discussing evidence regarding the level of implementation and impact of Canadian DPA policies.

### Policy content

Schools face many barriers to implementing DPA, and therefore the motivation to implement DPA may in some cases relate to the strength of accountability mechanisms in place to monitor implementation [116]. For this reason, provisions for monitoring and enforcement were used to distinguish strong from moderately strong policies [50]. None of the DPA policies met the benchmark for a strong policy, although policies in ON, BC (grades 10–12) and MB were rated as moderately strong. ON’s 2005 policy was the most prescriptive and specific, and might therefore be considered the strongest according to the criteria used. Policies in AB and BC (kindergarten to grade 9) were weak because they did not specify that PA should be performed at a moderate-to-vigorous intensity. The 2010 SK policy was perhaps the weakest of the 5, as it used non-specific language and gave School Boards the responsibility to develop their own DPA policies consistent with certain minimum standards. Moreover, although not described as optional within government policy documents, the policy was characterized as optional during discussion with government officials (Government of Saskatchewan, personal communication, September 4, 2014). It is therefore apparent that diffusion of Canadian DPA policies did not lead to a strengthening of policy over time. By comparison, among 14 US states with school-based DPA policies, 10 policies were weak, 4 were moderately strong, and none were rated as strong, using the same criteria [50]. Stronger state-level PE laws have been associated with stronger district-level PE policies [40], greater PE time allotments in schools [26], as well as increased PE attendance and PA among children [28], suggesting that strengthening the provisions of DPA policies, particularly provisions for monitoring and enforcement, is an important priority for policy makers.

Flexible delivery models are a hallmark of Canadian DPA policies, such that individual schools, teachers and students have been accorded substantial autonomy in determining the format in which PA is to be provided in schools. When adhered to, flexible delivery of PA opportunities positively impacts children’s PA and health outcomes [89-91,117]. Flexibility in implementation was important in minimizing initial resistance to DPA policies in AB [56], likely because it allowed DPA to take place with minimal displacement of time normally dedicated to academic pursuits. Flexible PA delivery can also reinforce the message to children that PA can be incorporated throughout their daily activities, and in a variety of locations. These schemes are also open to abuse, however, as they can lead to inconsistent and/or suboptimal policy implementation [118], and in BC led to falsification of student PA records in some cases [119]. Moreover, they may complicate attempts to monitor and enforce compliance since it may be less clear what constitutes ‘proper’ implementation.

A strong and consistent international body of knowledge regarding the positive benefits of PA for child health existed prior to the emergence of Canadian DPA policies, and all provinces cited this evidence as a rationale for their DPA policies. However, whereas evidence may have informed the decision to adopt DPA policies, it is less clear the extent to which evidence informed the specific provisions of each one. Some policies coincided with national recommendations at the time they were developed (e.g. the 2002 Canadian PA Guidelines for Children and Youth focused on MVPA and allowed short bouts of activity), while others did not (e.g. some policies did not focus on MVPA and required sustained PA) [120,121]. Moreover, given that policies covered only up to one third of the 90 minutes of PA children were recommended to accumulate at that time [120,121], children would have had to obtain the majority of their PA outside of school hours. Similarly in the US, only 35% of the PE-related legislation enacted between 2000 and 2007 contained one or more evidence-based statements [122]. Scientific evidence is only one factor policymakers consider during their deliberations [123,124], and thus these other factors may have superseded evidentiary concerns in some cases.

### Policy context

#### Adoption and diffusion

At a broad level, policy diffusion is the phenomenon whereby one government’s policy decisions are influenced by another’s [39]. Given the interconnected nature of policy networks, policy diffusion is a relatively common phenomenon, and one that is structured by the characteristics and capacities of adopters, their political contexts and the nature of the policies themselves [39]. In the case of DPA policies in Canada, patterns of adoption are consistent with 3 small ‘waves’ of adoption, with AB and ON leading the way, followed by MB and BC, and later SK. Notably, however, each province introduced a somewhat novel policy. Thus, although the *concept* of DPA policies diffused among some Canadian provinces, it is not clear to what extent the *content* of their respective policies diffused. This finding is consistent with the notion of re-invention, or the degree to which an innovation evolves during the course of its adoption and implementation [45]. Re-invention acknowledges that innovations are not fixed entities, nor are adopters passive recipients of new ideas [45]. Instead, adopters purposefully interact with, and actively modify new ideas to better fit their contexts [45]. In the current instance, considerable re-invention occurred as adoption proceeded, which may reflect factors such as contextual tailoring of policies or learning from the experiences of earlier adopters [125,126]. Thus, although they are not recognized as chronological innovators, later adopters did exhibit innovative behaviors through re-inventing earlier policies.

Adopter characteristics are among the most important determinants of diffusion. Earlier adopters tend to be more cosmopolitan, are more willing to take risks, are larger and better resourced, and have more extensive social networks [37,45]. Moreover, geographical diffusion models suggest distance is a critical factor, as policy adoption tends to occur later in more distal locations [38,126]. On a broad level, these qualities describe the adopters in this study, as adopters were geographically clustered within central and western Canada and, with the exception of Quebec, were the most populous and affluent in the nation (although Quebec is also large, relatively affluent and centrally located, in the interests of preserving its cultural heritage Quebec often acts independently of the rest of Canada) [106,107]. Implementation of DPA policies required additional human, financial and material resources that were already constrained within schools, and thus, notwithstanding the exceptional circumstances of Quebec, it is not surprising that the first 5 provinces to adopt DPA policies were the relatively affluent and most populous provinces that could better cope with risk and which, by virtue of their location, maintained closer connections with each other. By contrast, later adopters tend to be more skeptical of new ideas and require stronger evidence of success prior to adoption [45]. For this reason, subsequent ‘waves’ of DPA policy adoption may not occur until evidence substantively demonstrates policy success via positive change in health or other outcomes.

The concept of innovativeness refers to whether an adopter is early in adopting an innovation relative to other members of a social system [45]. Although Diffusion of Innovations theory sets forth 5 adopter categories, the framework acknowledges that innovativeness is more appropriately conceived as a continuous variable, with no sharp breaks between adopter categories [45]. Moreover as we have indicated, policy strength and implementation can vary substantially within adopter categories, potentially contributing to important variations in policy outcomes. Therefore, the current classifications represent simplifications based on theoretical constructs, and may not be optimal. For instance, although BC did not adopt DPA policies until 2008, the province piloted and widely disseminated an internationally recognized, successful model of DPA as early as 2003 through Action Schools! BC [88-91,96], and was the first province to mandate DPA at the high school level. BC was also among the first to develop nutrition guidelines for schools and recreational facilities, to develop a fruit and vegetable program for schools, and to institute a children’s fitness tax credit [127-129]. Thus, although it was slower to commit to a DPA policy than some other provinces, the province of BC may nevertheless be more innovative with respect to child health than the current classification suggests. Future studies could integrate other frameworks to provide a more nuanced perspective of adopter characteristics.

Diffusion of one type of policy can also stimulate diffusion of other related policies, and at other levels. In Canada, the proportion of schools with daily PE policies increased from 35% in 2006 to 55% in 2011 [130]. Compared to the national average, in 2011, schools in the western provinces and ON (i.e. DPA adopters) were more likely to have daily PE policies, and schools in the west were more likely to have fully implemented these policies [130]. These geographic and temporal trends suggest that DPA policies may have contributed to diffusion of daily PE policies at the school level, as schools attempted to comply with provincial DPA policies. Evidence of diffusion of PE policies from the state to the school district level has also been documented in the US [27,40].

Although this study focused on diffusion of policies within a Canadian context, examination of international trends is also relevant. Introduction of DPA policies in Canada coincided with a concurrent increase in school-based DPA policies in the US, with 4 policies enacted in 2005, 4 in 2006–08 and 6 in 2009–11 [50]. Although not captured in the previous analysis, in 2001, the Texas Legislature passed a bill requiring elementary school children to participate in 30 minutes of DPA or 135 minutes of PA weekly [131]. Given these concurrent trends, it is likely that consideration of broader international patterns of DPA policy diffusion is also warranted, as elements of Canadian and US policies, in particular, likely diffused across national borders.

In addition to findings regarding the conditional nature of diffusion, this analysis highlights the reality that policy adoption and diffusion are processes that unfold over the course of many years. As early as 1975, the MB Physical Education Working Group proposed that all MB schools be required to offer an average of 40 minutes of PE daily, including 20 minutes of vigorous PA [63], while the concept of Quality Daily PE, including provision of daily PA, was endorsed at a federal level in the late 1980s [15]. That the first DPA policy was not adopted until 2003, and that at present only 5 of Canada’s 13 provinces/territories have implemented DPA policies exemplifies the lengthy nature of policy cycles, which can span several decades [38,132]. In all cases of policy adoption it was not a single event, but rather a series of related events that unfolded over time and built momentum for change that ultimately led each province to develop DPA policies.

### Policy evaluation: Discussion of the systematic review

Policy evaluation is essential to justify ongoing implementation and can inform critical adjustments to improve policy impact. With the exception of SK, all of the provincial DPA policies have undergone some form of formal evaluation, as described below.

#### Implementation

The impact of school-based DPA policies ultimately depends upon the level of, quality and fidelity of implementation, however it is difficult to evaluate the extent of policy implementation due to the variety of measures used across studies and the flexibility inherent within each of the policies. At the school-level, ≥ 80% of ON schools reported taking action with respect to DPA policy implementation (defined as meeting the recommendations in several, but not all areas), however few (≤ 20%) reported consistently meeting or exceeding recommendations [133,134]. At the teacher-level in ON, only 16% reported always conducting DPA on days when PE was not scheduled [135], while at the student-level, 49% received DPA each school day [136]. The number of schools included in each of these studies was small, however, ranging from 18 to 37. Among 476 schools surveyed in BC, self-reported implementation of DPA policies was 65%, 56% and 51% for grades 6, 8 and 10, respectively [137]. Thus, data are consistent with moderate, but inconsistent implementation of DPA policies in schools, possibly as a result of the low to moderate strength of provincial-level policies and the lack of monitoring. Variation in implementation has the potential to worsen health disparities if better-resourced schools implement DPA policies to a greater extent, and thus assessment of the level of DPA implementation and developing strategies to improve compliance remain ongoing priorities.

A consistent finding across studies that have examined implementation of DPA policies in Canada [66,119,135], and provision of PA/PE in schools in general [17,23,89,118,138-144], is that PA-related goals may be difficult to achieve due to the additional time required to implement them. The use of standardized testing to assess the quality of educational instruction and student achievement has led to a prioritization of teaching in academic subjects [21,23,131,139]. According to Amis et al. [23], this culture of ‘academic achievement’ has led to a marginalization and de-valuing of PE in schools, and may in large measure be responsible for suboptimal implementation of school-based PE/PA policies. In light of overburdened school schedules, it is particularly problematic that DPA is often unscheduled, unstructured and not monitored [135,138]. In this context, DPA may easily be displaced in favor of academic pursuits that educators may perceive to be higher priority [23]. Integrating DPA into the curriculum and providing supportive resources, clear expectations and performance standards might help to avoid this outcome, such that PA becomes a normative and valued aspect of the school day [17]. Moreover, given that PA does not compromise, and may even improve academic performance among school-aged children [31-35], positioning DPA as a strategy to improve student academic achievement may increase support for policy adoption and implementation.

#### Impact

In ON, findings of a positive relationship between frequency of DPA and objectively measured overall PA and minutes of MVPA among elementary students in a very small sample of 18 schools are encouraging, however no child achieved the policy goal of ≥ 20 minutes of sustained MVPA, with the average PA bout lasting just 7.1 minutes [136]. The efficacy of the policy was, however, supported by findings showing that students who participated in DPA 5 days/week achieved higher total PA, the intensity of their PA was greater, and they accumulated more minutes of MVPA on school days and during the school day compared to students who participated in DPA < 5 days/week [136]. These results differ from a subsequent paper from the same study which showed that students in schools where administrators reported being in the ‘action’ phase of DPA implementation did not spend more time in light-to-vigorous PA compared to students from schools in the ‘initiation’ phase [133]. Similarly, implementation of DPA in ON was not associated with student self-reported PA or BMI in 30 elementary schools [134,145-147]. Differences across studies likely relate to the various exposure and outcome variables considered, and the small number of schools examined.

Findings in MB of a decline in PA as students progressed from grades 9–12 following implementation of the MB PE/Health Education curricula (including DPA for grades 11–12) are similarly difficult to interpret [148]. Given that PA levels decline as children transition to adolescence and adulthood [149-155], it is possible that MB’s PE policy slowed this decline, however, as there was no control group, it is not clear what the students’ PA trajectories would have been in the absence of the policy. Furthermore, the study was conducted in the context of a provincial PE/Health Education policy which only mandated DPA for students in grades 11 and 12 in the form of 55 hours of self-reported MVPA in each of 2 semesters. Thus, an increase in PA would not necessarily be expected in this context, and findings may point to important limitations of the policy. The slower rate of decline in PA among children attending low socioeconomic status schools is noteworthy, and suggests the MB policy may ameliorate disparities in PA [148].

In BC, schools were more likely to meet the recommended amount of 150 minutes/week of PE for grade 6 students, and to provide more minutes of PE to grade 8 and 10 students following implementation of DPA policies, however the impact on children’s PA levels was not assessed [137]. Positive impacts of Action Schools! BC on student PA and health outcomes have already been demonstrated [89-91], and thus it will be important to determine whether BC’s DPA policy provides additional benefits. Impact of the AB and SK policies has not been assessed, however data from the Canadian Fitness and Lifestyle Research Institute [114] suggest that provincial DPA policies have not significantly increased school-aged children’s PA levels in any of the Canadian provinces. Notably, however, these data represent the PA levels of all school-aged children, whereas most provincial DPA policies only apply to a subset of school-aged children.

Overall, findings indicate that Canadian DPA policies, as currently implemented, have had little to no impact on school-aged children’s PA levels or BMI; although given the paucity of studies and their limitations, it is too early to draw definitive conclusions as to their efficacy, or to distinguish among relatively less and more efficacious policies. These tentative findings must also be considered in light of the fact that policy implementation was moderate in the ON and BC-based studies, and that there may be substantial variation among schools in strategies used to implement DPA policies [119]. The time frame for assessment relative to policy implementation must also be considered, as the effects of policy may take many years or decades to materialize [38,132]. Forthcoming findings related to policy impact in ON may help to inform more robust conclusions in this respect.

These findings differ from those of a systematic review of school-based PA policies in the US. Similar to our study, outcomes for only a small number of policy reforms were reviewed, however in that review school-based PA policies appeared effective in increasing youth PA levels [36]. Importantly, of the 3 studies that assessed impact of PA policies, none used objective measures to assess change in children’s PA levels from pre- to post-policy implementation [36]. Differences among the populations examined (e.g. age, sex, socioeconomic status), in assessment tools and in specific outcomes examined must also be considered. A recent study that used objective measures to examine impact of a policy in Boston schools to promote 150 minutes/week of MVPA found that the policy led to a significant increase of 3.9 minutes/day of MVPA among elementary school children [156]. In addition, several studies that have embedded DPA within broader comprehensive school health initiatives have effectively increased children’s PA levels [91,157], suggesting that DPA policies might prove more effective when implemented within this context.

### Strengths and limitations

A multiple case history approach allowed us to study policy adoption and diffusion not as events, but as processes that unfolded over time, providing a rich perspective that could not have been achieved through quantitative, survey-based methodology. A systematic review of the evidence concerning DPA policies offered rich contextualization of the case histories presented. The thorough and extensive nature of our searches, follow-up of all relevant references, wide diversity in the types of documents examined, convergence of findings from different sources, and involvement of multiple reviewers lends credibility to the findings, and suggests we have captured a broad and diverse perspective of the factors that influenced adoption and diffusion of Canadian DPA policies. Nevertheless, this analysis was restricted to a review of web-based, publicly available documents, and thus we may have missed older documents less likely to be posted on the internet. The history of DPA legislation is relatively recent, however, and we were still able to identify historically influential documents through reference lists. The number and type of documents available in each of the provinces was beyond our control, and may have influenced our findings. We focused on retrieving documents at a provincial level, however activities at other levels were also likely influential, but were outside the scope of the current analysis. We did not evaluate the quality of the studies included in the systematic review as there are no tools capable of providing a fair assessment of the quality of natural experiments [36]. Although generalizability is unclear, these case studies provide contextual details useful for assessing generalizability of findings to other contexts.

Although our case histories showed how DPA policies progressed from conception to implementation, it was often not possible to discern the rationale for specific policy provisions (e.g. why MB only mandated DPA for grades 11–12). Review of internal documents and/or interviews with key stakeholders may provide additional insight into the processes surrounding adoption and diffusion of DPA policies, and we are currently conducting key informant interviews in this respect. These data may uncover other influential factors less likely to be discussed in documents, such as the role of policy champions. Conversely, key informants may not be fully aware of the rich and extensive history of efforts to embed health promotion within schools in their respective jurisdictions, and therefore together these analyses will provide a comprehensive and compelling summary of the processes underlying adoption and diffusion of DPA policies in Canada. Given that Diffusion of Innovations theory focusses primarily on the characteristics of innovations and adopters rather than on their political, social and economic contexts, additional explanatory frameworks may be integrated to describe these data.

## Conclusion

This is the first study to collate comprehensive national data with respect to adoption, diffusion, implementation and impact of school-based DPA policies. This study detailed the history and current status of Canadian DPA policies, highlighting the conditional nature of policy adoption and diffusion, and describing policy and adopter characteristics and political contexts that shaped policy trajectories. Findings point to key levers that may have led to some provinces being relatively earlier in adopting DPA policies. An understanding of the conditions associated with successful policy adoption and diffusion can help to identify receptive communities and contexts in which to pioneer novel legislative solutions to the problem of inactivity among children, and is critical to inform efforts to catalyze and accelerate diffusion of health promoting policies in Canada and internationally. However, ensuring implementation of efficacious DPA policies is as important as adopting them. By reviewing evidence regarding implementation and impact of such policies, this study can inform useful amendments to existing policies, and help to strengthen provisions within future legislation. Findings also point to specific gaps within the currently available literature that provide direction for future studies.

Policy making is admittedly a controversial and complex endeavor. Current PA practices in schools are the product of a myriad of policies enacted over several decades. Thus, it may take decades to reverse entrenched policies that bound PA in the school environment, and to establish school communities in which PA is a normative and valued aspect of the school day. Adoption and diffusion of efficacious and effective policies are essential first steps in reshaping PA-related social norms in health-promoting directions. However, the potential of DPA policies is currently constrained by an education system that focuses strongly on academic achievement. To overcome these and others barriers, provinces should integrate DPA within current curricula and monitor policy implementation. Furthermore, the potential benefits of PA on academic outcomes should be emphasized. Investigation of adoption, implementation and impact of DPA policies remains an ongoing priority, both to inform policy development, and because sound evidence of policy efficacy and effectiveness can accelerate policy diffusion. Such studies should ideally be structured as natural experiments. Policy, however, is not a panacea, and school-based DPA policies alone will not be sufficient to enable children to meet PA recommendations. Opportunities for intervention are numerous, and thus policies must be complemented by dietary and other PA-related interventions in a variety of settings.

## Additional file

Additional file 1:
**Database search strategy for systematic review.**
