# Supplementary material for: A multiple case history and systematic review of adoption, diffusion, implementation and impact of provincial daily physical activity policies in Canadian schools
Source: BMC Public Health. 2015 Apr 15;15:385. doi: 10.1186/s12889-015-1669-6 (PMC4436021; doi:10.1186/s12889-015-1669-6)
Supplement: Additional file 1: — Database search strategy for systematic review. [file 12889_2015_1669_MOESM1_ESM.docx]

**LITERATURE SEARCH—*Physical Activity Policies in Schools***

Limits: English and French, published between 01/01/2003 – current

Database: Ovid MEDLINE(R) In-Process & Other Non-Indexed Citations and Ovid MEDLINE(R) 1946 to Present

| 1. Health Policy/  2. Government Programs/  3. exp Policy Making/  4. legislation & jurisprudence.fs.  5. (law or laws).tw.  6. legislat*.tw.  7. memorand*.tw.  8. (policy or policies).tw.  9. or/1-8 [450391 - policy terms]  10. Schools/  11. Students/  12. (elementary adj2 school*).tw.  13. (grade* adj5 school*).tw.  14. high school*.tw.  15. (middle adj2 school*).tw.  16. (primary adj2 school*).tw.  17. (secondary adj school*).tw.  18. or/10-17 [86135 – school terms]  19. exp Motor Activity/  20. (active adj (commut* or transportation)).tw.  21. exercis*.tw.  22. physical activit*.tw.  23. or/19-22 [365048 – physical activity terms]  24. 9 and 18 and 23  25. (animals not (animals and humans)).sh.  26. 24 not 25  27. limit 26 to (yr=”2003 – Current” and (english or french)) [470 – school, policy, physical activity terms]  28. meta analysis.mp,pt.  29. review.pt.  30. search*.tw.  31. or/28-30  32. 27 and 31  33. remove duplicates from 32 [37 - review filter] |
| --- |

Database: Ovid PsycINFO 2002 to July Week 5 2014

| 1. Government Programs/  2. exp Policy Making/  3. (law or laws).tw.  4. legislat*.tw.  5. memorand*.tw.  6. (policy or policies).tw.  7. or/1-6 [112435 - policy terms]  8. Schools/  9. Students/  10. (elementary adj2 school*).tw.  11. (grade* adj5 school*).tw.  12. high school*.tw.  13. (middle adj2 school*).tw.  14. (primary adj2 school*).tw.  15. (secondary adj school*).tw.  16. or/8-15 [78143 – school terms]  17. exp Physical Activity/  18. (active adj (commut* or transportation)).tw.  19. exercis*.tw.  20. physical activit*.tw.  21. or/17-20 [42215 – physical activity terms]  22. 7 and 16 and 21 [260 – school, policy, physical activity terms]  23. (animals not (animals and humans)).sh.  24. 22 not 23  25. limit 24 to (yr=”2003 – Current” and (english or french)) [253 – school, policy, physical activity terms]  26. meta analysis.mp,pt.  27. review.tw.  28. search*.tw.  29. or/26-28  31. 25 and 29 [24 – review filter] |
| --- |

Database: Ovid ERIC 1965 to June 2014

| 1. exp administrative policy/  2. educational legislation/  3. educational policy/  4. federal government/  5. federal legislation/  6. federal programs/  7. federal state relationship/  8. government role/  9. exp government school relationship/  10. laws/  11. legislation/  12. national programs/  13. policy/  14. policy analysis/  15. policy formation/  16. exp public policy/  17. school law/  18. school policy/  19. state legislation/  20. state government/  21. state policy/  22. state programs/  23. state regulation/  24. state school district relationship/  25. states powers/  26. statewide planning/  27. (law or laws).tw.  28. legislat*.tw.  29. memorand*.tw.  30. (policy or policies).tw.  31. or/1-30 [225596 - policy terms]  32. elementary school students/  33. elementary schools/  34. magnet schools/  35. middle school students/  36. middle schools/  37. public schools/  38. rural schools/  39. secondary school students/  40. secondary schools/  41. schools/  42. urban schools/  43. elementary school*.tw.  44. grade school*.tw.  45. (high school* or highschool*).tw.  46. middle school*.tw.  47. primary school*.tw.  48. secondary school*.tw.  49. or/32-48 [281256 – school terms]  50. Physical Activities/  51. (active adj (commut* or transportation)).tw.  52. physical activit*.tw.  53. or/50-52 [6400 – physical activity terms]  54. 31 and 49 and 53 [175 – school, policy, physical activity terms]  55. limit 54 to (yr=“2003 - Current” and (english or french)) [141 – school, policy, physical activity terms]  56. (meta analys* or metaanalys*).mp.  57. review*.tw.  58. overview*.tw.  59. search*.tw.  60. or/56-59  61. 55 and 60 [14 – review filter] |
| --- |

Database: SPORTDiscus with Full Text via EBSCOhost

| 1. (law or laws)  2. legislat*  3. memorand*  4. (policy or policies)  5. S1 OR S2 OR S3 OR S4 [73,189 – policy terms]  6. DE “SCHOOLS”  7. DE “SCHOOLS – Exercises & recreations”  8. DE “SCHOOL children”  9. DE “STUDENTS”  10. DE "STUDENTS -- Recreation"  11. (elementary N2 school*)  12. (grade* N5 school*)  13. “high school*”  14. (middle N2 school*)  15. (primary N2 school*)  16. (secondary N1 school*)  17. S6 OR S7 OR S8 OR S9 OR S10 OR S11 OR S12 OR S13 OR S14 OR S15 OR S16 [50,997 – school terms]  19. DE "PHYSICAL activity"  20. (active N1 (commut* or transportation))  21. “physical activit*”  22 S18 OR S19 OR S20 [38,670 – physical activity terms]  23. S5 AND S17 AND S21 [292 – school, physical activity, policy terms]  24. S5 AND S17 AND S21 Limiters - Published Date: 20030101-20141231; Language: English, French [236]  25. (“meta analys*” or metaanalys*)  26. review  27. search*  28. S24 OR S25 OR S26 [review]  29. S23 AND S27 [14] |
| --- |
